# Monitoring Rarity: The Critically Endangered Saharan Cheetah as a Flagship Species for a Threatened Ecosystem

**DOI:** 10.1371/journal.pone.0115136

**Published:** 2015-01-28

**Authors:** Farid Belbachir, Nathalie Pettorelli, Tim Wacher, Amel Belbachir-Bazi, Sarah M. Durant

**Affiliations:** 1 Institute of Zoology, Zoological Society of London, London, United Kingdom; 2 Department of Anthropology, University College London, London, United Kingdom; 3 Laboratoire d’Ecologie et Environnement, Université de Béjaïa, Béjaïa, Algeria; 4 Conservation Programmes, Zoological Society of London, London, United Kingdom; 5 Wildlife Conservation Society, Bronx, New York, United States of America; University of York, UNITED KINGDOM

## Abstract

Deserts are particularly vulnerable to human impacts and have already suffered a substantial loss of biodiversity. In harsh and variable desert environments, large herbivores typically occur at low densities, and their large carnivore predators occur at even lower densities. The continued survival of large carnivores is key to healthy functioning desert ecosystems, and the ability to gather reliable information on these rare low density species, including presence, abundance and density, is critical to their monitoring and management. Here we test camera trap methodologies as a monitoring tool for an extremely rare wide-ranging large felid, the critically endangered Saharan cheetah (*Acinonyx jubatus hecki*). Two camera trapping surveys were carried out over 2–3 months across a 2,551km^2^ grid in the Ti-n-hağğen region in the Ahaggar Cultural Park, south central Algeria. A total of 32 records of Saharan cheetah were obtained. We show the behaviour and ecology of the Saharan cheetah is severely constrained by the harsh desert environment, leading them to be more nocturnal, be more wide-ranging, and occur at lower densities relative to cheetah in savannah environments. Density estimates ranged from 0.21–0.55/1,000km^2^, some of the lowest large carnivore densities ever recorded in Africa, and average home range size over 2–3 months was estimated at 1,583km^2^. We use our results to predict that, in order to detect presence of cheetah with p>0.95 a survey effort of at least 1,000 camera trap days is required. Our study identifies the Ahaggar Cultural Park as a key area for the conservation of the Saharan cheetah. The Saharan cheetah meets the requirements for a charismatic flagship species that can be used to “market” the Saharan landscape at a sufficiently large scale to help reverse the historical neglect of threatened Saharan ecosystems.

## Introduction

Deserts cover more than 17% of the world’s land mass [1], yet are among the most poorly understood biomes. Their relatively poor productivity, driven by low rainfall, supports low abundances of wildlife, and hence they tend not to attract the attention of conservationists [2], particularly in the current global focus on biodiversity hotspots [3,4]. Yet those species harboured by arid biomes are often of particular significance, as their resilience to water scarcity can provide key insights into evolution and genetics that may have great potential to agriculture and conservation [5]. Moreover, despite the harsh conditions, deserts harbour high biodiversity levels, supporting 25% of global terrestrial vertebrates [1]. However, a lack of research and conservation focus, coupled with increased anthropogenic pressure and high susceptibility to climate change [6,7], threaten desert ecosystems, and render many desert species particularly vulnerable to extinction [2,3].

Historically, deserts were dominated by pastoralist communities that were nomadic and able to move in response to local and highly variable rainfall [8–10]. However, in recent years, these communities have been under increasing pressure to settle. Changes in land use and tenure policies are often incompatible with nomadic lifestyles, while provision of permanent water and capital investment for agricultural development provides incentives to stay in one place [11]. In many areas these interventions have led to increased overgrazing and desertification, leading to further constraints on resources, with potentially catastrophic results for wildlife and people [12]. The impacts of climate change are likely to further aggravate these pressures [11].

In low rainfall environments, large herbivores typically occur at low densities, and their large carnivore predators at even lower densities [13]. However, the continued survival of large carnivores is key to the healthy functioning of ecosystems [14]. For example, the disappearance of predators can lead to an increase in herbivores, and knock on impacts on tree and shrub regeneration [15]. Degradation of grazing resources, especially if combined with overhunting, will result in a reduction in wild prey for large carnivores, which can lead to an increase in depredation of livestock by carnivores [16]. The ability to monitor large carnivores and their ecology in the face of these anthropogenic pressures will be key to understanding the resilience of carnivore populations to increasing impacts of anthropogenic environmental change.

Monitoring is also key to sustainable management of desert ecosystems that maintains productive habitats able to support populations of large herbivores and viable populations of top predators. Monitoring enables evaluation of different management and policy interventions, understanding the impacts of anthropogenic change, and taking action to prevent the loss of species. This is particularly pertinent in the case of large carnivores in harsh and highly variable desert environments, which are likely to be especially scarce and hence their detectability extremely low. At present, there are limited survey and monitoring tools available for species which occur at extremely low densities. Yet reliable methods to confirm continued presence, monitor density, and develop an understanding of the ecology of extremely rare, low density carnivores is key to their future conservation, and hence to securing the survival of functioning desert ecosystems. Of particular importance are sampling recommendations to enable the design of cost effective surveys that deliver estimates of presence and abundance at sufficient power to be meaningful [17].

The Saharan cheetah (*Acinonyx jubatus hecki*) is a critically endangered large felid now largely confined to desert environments [18], whose range is limited to pockets in north and west Africa, and which is thought to number less than 250 individuals [18,19]. This enigmatic and rare large carnivore has been recently documented in the Ahaggar Cultural Park (ACP) in south central Algeria [20–22] yet virtually nothing is known about the subspecies. Limited genetic analyses conducted to date support the genetic distinctiveness of the Saharan cheetah [23].

Here we use information from two camera trapping surveys carried out in the Central Saharan Ahaggar massif located in south central Algeria to examine the ecology of the Saharan cheetah for the first time. Desert environments are characterised by poor availability of resources, such as water and cover, often needed for large carnivores to hunt successfully [24], and an unpredictable rainfall that results in nomadic behaviours of potential ungulate prey [25,26]. Furthermore, the Sahara is subjected to hot summers, with day-time temperatures regularly in excess of 40°C. These factors may place additional pressures on a species such as the cheetah, whose hunting strategy depends on attaining speeds at the limits of performance [27], and which may suffer energetic constraints imposed by large travel distances [28]. Based on this, we therefore hypothesize that cheetah in the Sahara will be constrained behaviourally and ecologically relative to more productive savannah ecosystems. In particular, we predict that cheetah in the Sahara compared with those in more productive ecosystems will (P1) avoid constraints imposed by extreme heat by shifting to nocturnal activity; (P2) range more widely; and (P3) live at lower densities. We use our data to test these predictions and go on to make recommendations for future surveys to confirm presence and estimate abundance.

## Methods and Materials

This research was non-intrusive using remote infra-red camera traps, and is unlikely to have caused any disturbance to the wildlife. The project was carried out under a research permit issued to Farid Belbachir by the Algerian Ministry of Culture’s Direction de la Protection Legale des Biens Culturels et de la Valorisation du Patrimoine Culturel (Directorate of Legal Protection of Cultural Property and the enhancement of cultural heritage). No specific permissions were required under this permit for the use of remote camera traps. The project had the full support of the parks authority, who frequently accompanied the survey team, and helped provide backup vehicles and logistical support.

### Study area

**Figure 1:**
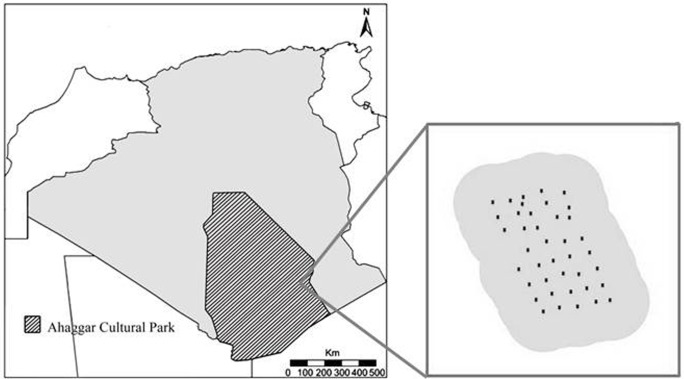
Study area, detailing the location of the study area and the surveyed region.

The study was carried out over two field seasons (August-October 2008 and August-November 2010) in the ACP; a very large protected area covering 633,887km^2^, located in south central Algeria, and belonging to the administrative department (wilaya) of Tamanrasset [29]. The ACP was officially established as Parc National de l’Ahaggar in 1987. It is managed by Office National du Parc Culturel de l’Ahaggar (ONPCA) and falls under the Ministère de la Culture [29]. The park is managed to protect, conserve and enhance cultural and natural heritage, and is designated to have category II protection in the world database of protected areas (www.protectedplanet.net). The park is occupied by local Tuareg communities, who are traditionally semi-nomadic pastoralists, and maintain herds of camels and smallstock. Today many are permanently settled in small settlements centred in areas with access to water, and with limited areas of agriculture. The survey area encompassed a region where cheetah tracks and signs were found, and scats collected, during a previous survey [20,22] (Fig. 1).

The Ahaggar is a Precambrian crystalline mountainous region with a highly variable topography; reaching 3,000m at the Mount Atakor. This region is characterized by the presence of deep valleys and depressions, harbouring temporary and permanent gueltas (water pools), with wider vegetated wadis (valleys) supporting patches of *Tamarix* spp. and *Acacia* spp., and with some stony plains and limited dune areas [30]. The bioclimate ranges from hyper-arid to sub-arid, and meteorological conditions are highly variable, with annual rainfall ranging between 20–100 mm. Temperatures can range between −7°C and 50°C, depending on altitude and season [30]. The mammalian biodiversity is little known, however cheetah, African wildcat (*Felis silvestris lybica)*, Rüppell’s fox (*Vulpes rueppellii)*, and golden jackal (*Canis aureus)* were found in an earlier survey [31]. The sand cat (*Felis margarita)* and Fennec fox (*Vulpes zerda)* were recorded during the course of this study in 2008 [32], and leopard (*Panthera pardus)* was recently recorded in Ti-n-hağğen [20,31]. Barbary sheep (*Ammotragus lervia*) and dorcas gazelle (*Gazella dorcas*) are present, as are other wild herbivores, including Cape hare (*Lepus capensis)*, rock hyrax (*Procavia capensis)* and M’Zab gundi (*Massoutiera mzabi)*. Livestock is represented by free ranging and hobbled dromedaries (*Camelus dromedarius)* and feral asses (*Equus africanus)*; whereas smallstock—mainly goats, mixed with proportionally fewer sheep—are closely herded.

The main economic and human activities in the study area are nomadic and semi-nomadic pastoralism and desert tourism. Natural vegetation constitutes important forage for both wildlife and livestock. Tourism was negatively hit by security issues prevailing in the region during the 1990s because of political instability affecting the country at that time [30], and numbers of tourists remained low during this study.

### Survey design and field sampling

A rectangular trapping grid was designed and overlaid on a satellite image of the study area using 40 camera trap locations, spaced 10 km apart, covering a total area of 2,551 km^2^. The survey used fixed Reconyx (model RC55) cameras, which use passive infra-red motion detectors that trigger a photograph when animals pass in front of the camera. A 10km spacing distance was chosen as a compromise to: (1) ensure total area covered was sufficiently extensive to encompass more than a single cheetah monthly home range—cheetah have been documented to have average annual home ranges ranging up to 1,651km^2^ (Table 1); (2) to ensure cameras were sufficiently close together so as no home range was missed between camera placement. Only the unusually small territories of male cheetah in the prey rich Serengeti have been documented to be smaller than 100km^2^. It is anticipated that, in desert environments, where prey density is low, cheetah home range size is likely to be closer to the maximum documented.

**Table 1:** Published estimates of home range size and population density of cheetah. Where information is available, home range is separated into territorial and non-territorial cheetah.

Location	Density (no. of individuals per 100 km^2^)	Home range (km^2^)		Reference
		Territorial	Non territorial	
Namibian farmlands	0.25–0.83	1716		Marker 2002 [53]; Marker et al. 2008 [56]
Kruger National Park, South Africa	0.88	126	195	Bowland 1995 [54]; Broomhall et al. 2003 [71]
Serengeti National Park, Tanzania	2.5	48	777–833	Caro 1994 [49]; Durant et al. 2011 [55]
Ahaggar Cultural Park	0.023	1583		This study

Cameras were usually placed under the nearest tree within 1km of each pre-allocated grid point. Trees were selected as they were likely to be attractive to passing cheetah; however they had the added advantage of providing shade for the camera traps, protecting them from the heat of the day. Single cameras were set to rapid fire (photos taken rapidly one after another for as long as the camera’s detector is triggered) to enable animals attracted to trees to be repeatedly captured as they moved around, thus allowing both sides to be photographed. Cameras were usually attached to tree bases, but sometimes were attached to wooden planks or rocks, at circa 40cm height. No bait was used. All trap stations were georeferenced using a GPS receiver (Model Garmin eTrex Venture HC). Camera devices were monitored once every two weeks to replace alkaline batteries and download data to a laptop computer (from 4 gigabyte-memory cards). Setting up the first survey took 17 days, and the second survey 8 days. Cameras were removed in the same order they were set up, and were continuously active over a total duration of 2 months (13 August–13 October) and 3 months (24 August–22 November) in 2008 and 2010, respectively.

### Data analysis

The time of day of cheetah captures recorded by the camera traps was examined to determine whether cheetah captures were associated with day or night. In order to test whether any observed nocturnal activity was associated with the availability of moonlight, nocturnal captures were further examined against the phase of the moon using a chi-squared goodness of fit test. For this analysis, first and last quarter moon phases were classified as dark, and the second and third quarters as bright. Individual cheetah were identified from photographs by their unique spot patterns. Spot patterns from three different regions from any part of each animal’s pelage were considered for comparison between cheetah photographs; allowing unambiguous individual recognition [33]. An ‘x matrix’ of capture histories [34,35] of individual cheetah photographed was built for each of the 2008 and 2010 surveys. CAPTURE was used for abundance estimation. We were unable to explore spatial variability in detection probability due to low sample size. Sampling surveys were collapsed into 9 and 13 seven-day sampling occasions corresponding to 2008 and 2010, respectively.

Data were tested for violation of assumptions of closure, i.e. that the population is closed to immigration, emigration, mortality and reproduction, using CAPTURE [34,36,37]. Where violations in closure assumptions were detected, an alternative software, CloseTest, was used to compare a null model that allows for time-specific variation, such as temporary emigration, against an alternative hypothesis of a completely open population that allows mortality and recruitment [38,39]. Several models (the null model M_0_, heterogeneity model M_h_, behavioural response model M_b_ and time variation model M_t_, as well as different combinations of these models, M_bh_, M_th_, M_tb_, and M_tbh_) were compared using CAPTURE when estimating cheetah population size. Selection of the final model was based on examination of the results of the goodness-of-fit and between model tests (included in CAPTURE and developed by Otis et al. 1978 [34]). Capture probabilities and cheetah population size were estimated using the Jackknife estimator under heterogeneity model M_h_, and Chao’s estimator under time and heterogeneity model M_t_h [40], in 2008 and 2010 respectively.

To estimate cheetah densities, it was necessary to estimate the effective area sampled by the camera trap surveys. We did so by evaluating the size of the area delineated by the outermost camera traps and adding a buffer strip whose width is related to the mean maximum distance moved (MMDM) among multiple captures of individual cheetah within the 2008 and 2010 survey periods (see [35]). We present results for a buffer of half and full MMDM [41–44]. A coarse estimate of the average home range size of male cheetah in Ti-n-hağğen region over the survey period was obtained by using the mean maximum distance moved between captures of individuals, from the 2008 and 2010 surveys.

To develop recommendations for future surveys for low density, wide-ranging species we used our data to estimate the probability of detecting cheetah if survey effort was reduced. We did this in three ways:

- We plotted an accumulation graph of the number of individual cheetah captured during each survey as survey time increased, across all camera traps.
- We estimated the probability of detecting presence (i.e. one or more cheetah) from surveys of varying duration across all camera traps from our data as follows: For a survey of total duration *t* we extracted from our data each possible sub-survey of *t_s_* consecutive days and calculated whether at least one cheetah had been detected or no cheetah had been detected. The overall mean detection for a sub-survey of duration *t_s_* was calculated as the proportion of sub-surveys where at least one cheetah had been detected out of all possible sub-surveys.
- We estimated the probability of detecting presence from surveys of avarying number of camera traps across the duration of surveys as follows: For a survey of *n* camera traps we calculated the probability of detecting at least one cheetah (*P(n*)) using the following formula for Bernouilli trials:
  $$P\left(n\right)=1-{\left(1-p_{c}\right)}^{n}$$
  Where *p_c_* = the probability of detecting a cheetah at one camera station. *p_c_* was calculated from the number of camera stations where at least one cheetah was detected over the duration of the survey divided by the total number of camera stations.

For reporting purposes, survey effort was standardised to number of camera trap days—i.e. number of camera traps multiplied by number of days of survey.

## Results

Photographs were obtained from 15 captures of cheetah in 2008 and 17 captures in 2010. For all except one individual, a series of photographs were obtained for each cheetah, covering both sides of each individual. Only one sighting, in 2008, where it was impossible to identify the individual, was discarded from CAPTURE analyses. The data in 2008 and 2010 yielded captures of four adult cheetah (3 males and 1 of unidentified sex) and two adult cheetah (2 males) and one subadult (unidentified sex) respectively. Camera-trap effort totaled 1862 trap-days in 2008 and 3367 trap-days in 2010 (Table 2). Overall, an average of 124.1 and 198.1 trap-days were necessary to capture a single cheetah picture in 2008 and 2010, respectively.

**Table 2:** Sampling effort characteristics and camera-trap captures in Ti-n-hağğen region, Ahaggar Cultural Park, Algeria, 2008 and 2010.

Year	Sampling period	No. of camera stations	No. of camera trap days (24h)	No. photos	No. of cheetah captures	No. of individuals
2008	13/8–13/10	40	1862	302	15	4
2010	24/8–22/11	40	3367	493	17	3

**Figure 2:**
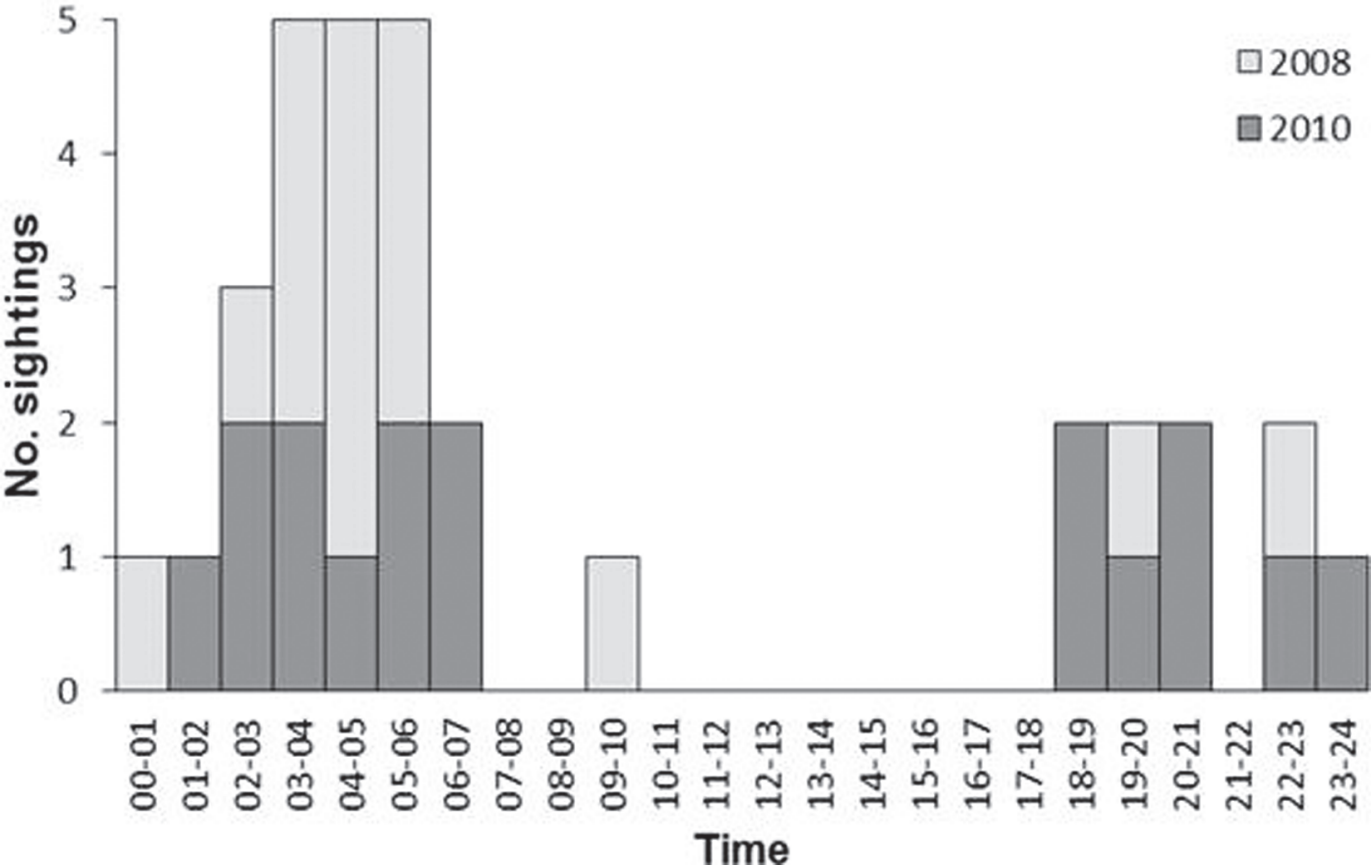
Time of cheetah captures across the two surveys in 2008 and 2010. Data covers 32 captures of 5 different individuals.

Of the 32 captures in total, 30 were captured after sunset (range 17:52–19:11 over course of surveys) and before sunrise (06:14–06:56). Nearly half (47%) of captures occurred in the small hours of the morning between 03:00–06:00 (Fig. 2). Of the two captures during daylight, only one was captured well after sunrise at 09:20, the other was only 12 minutes after sunrise. There was no evidence of captures being linked to the phase of the moon, since nocturnal captures were no more likely during the second and third quarters than from the first and last quarter of the lunar cycle (χ^2^ = 0.38; df = 1; p = 0.538).

Mean maximum distance travelled over both surveys, calculated from the maximum direct distance between camera trap locations of captures of individuals from whom five or more captures were received, was 44.9km (n = 2). Using this distance as a radius, and assuming an approximate circular home-range, average home range size over the 2–3 month survey period was estimated as 1583km^2^ (Table 1), and 100% MCP estimation yielded a maximum home range of 1,337km^2^. The use of half MMDM (22.45km) and full MMDM (44.9km) as a buffer results in effective survey areas of 9,029km^2^ and 19,069km^2^ respectively (Table 3).

**Table 3:** Population closure, sampling occasions, estimated population, densities from Ti-n-hağğen region, Ahaggar Cultural Park, Algeria.

Survey year	Sampling occasions	Final model	Capture probability (p̂)	Abundance ± SE	95% CI	Closure test		Effective area sampled (km^2^)	Density (no. individuals/1,000km^2^)
					*z*	*p*			
2008	9	M_h_	0.20	5 ± 1.36	5–11	−2.685	0.004	½ MMDM 9,029	0.55
								Full MMDM 19,069	0.26
2010	13	M_th_	0.21	4 ± 1.43	4–11	−0.250	0.401	½ MMDM 9,029	0.44
								Full MMDM 19,069	0.21

In 2008, the assumption of population closure was not met using the closure test in CAPTURE (Table 3), however, CloseTest showed no evidence for an open population (χ^2^ = 2.692; df = 3; p = 0.442) suggesting that the violations detected by CAPTURE may be due to temporary emigration or immigration [38,39]. Neither behavioural response after initial capture (M_0_ vs. M_b_; χ^2^ = 0.089; df = 1; p = 0.765) nor time-specific variation (M_0_ vs. M_t_; χ^2^ = 3.704; df = 8; p = 0.883) in trapping probabilities could be detected. M_0_ was identified as the best fit (score = 1.00) for the data; whereas M_h_ ranked the second highest with a score of 0.92. The heterogeneity model M_h_ was chosen over M_0_ (Table 3) due to: a non-significant goodness-of-fit test (χ^2^ = 12.857; df = 8; p = 0.117); a robust Jackknife estimator [45]; and known high levels of detection heterogeneity for cheetah elsewhere (e.g., [46]). Under this model, the Jackknife estimator produced an average capture probability p_1_ = 0.200 and an abundance estimate of 5 individuals (5 ± 1.36 [5–11, 95% CI]; CV = 27.18%) with all or part of their home ranges included in the survey area. This resulted in a population density estimate of 0.55 and 0.26 cheetah/1,000km^2^ in 2008 using a buffer of half and full MMDM respectively (Table 3).

In 2010, assumptions of population closure were met (Table 3). The time and heterogeneity model M_t_h was identified as the best fit to the data (score = 1.00). The remaining models ranked relatively low; with the time, behaviour and heterogeneity model M_t_bh being the second highest with a score of 0.72. Time-specific variation in trapping probabilities was confirmed during the 2010 sampling period by comparing between models M_0_ and M_t_ (χ^2^ = 37.625; df = 12; p<0.001). Average capture probability was estimated as p_2_ = 0.212, using Chao’s estimator. Four individuals (4 ± 1.43 [4–11, 95% CI]; CV = 35.7%) were estimated to roam in the survey area during the 2010 survey period, leading to an estimated density of 0.44 and 0.21 cheetah/1,000km^2^ using a buffer of half and full MMDM respectively (Table 3).

**Figure 3:**
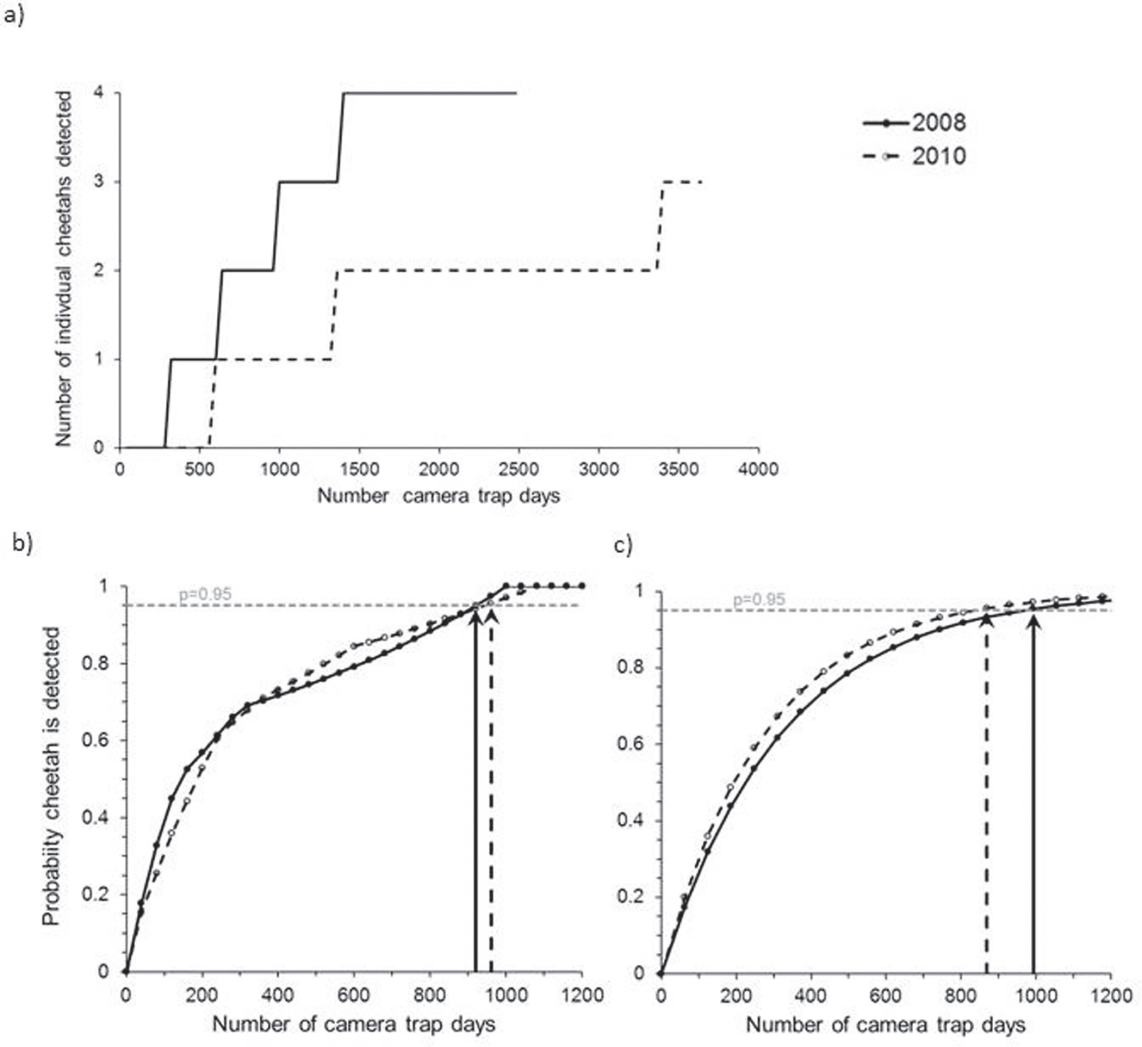
Survey effectiveness at delivering abundance and presence estimates with increasing survey effort measured in camera trap days (number of camera traps × number of days of survey). a) Accumulation graph of number of individuals captured during surveys; b) probability of detecting at least one cheetah with increasing effort, keeping the number of camera stations constant; and c) probability of detecting at least one cheetah with increasing effort, keeping the survey duration constant. The vertical lines in b) and c) depict the number of camera trap days that result in 95% probability of detecting presence.

While overall estimates of density were relatively similar in both surveys, the pattern of accumulation of identified individuals differed. In 2008 all four individual cheetah identified within the survey were recorded by day 35, after 1,400 camera trap days, whereas in 2010 it was not until day 82, after 3280 camera traps days, that all three individual cheetah identified over the survey were captured (Fig. 3A). However, both surveys predicted a similar effort needed to confirm the presence of cheetah. When using the 40 camera station survey design and varying the number of days, cheetah were detected in over 95% of consecutive samples of 23 days or more in 2008, and 24 days in 2010, corresponding to 920 and 960 camera trap days, respectively (Fig. 3B). When varying only the number of camera stations, cheetah were detected with over a 95% probability when 16 camera stations or more were deployed in 2008 and 14 in 2010, corresponding to 992 and 868 camera trap days, respectively (Fig. 3C). The longer survey duration in 2010 is likely to have contributed to a slightly lower number of camera trap stations needed to meet the threshold of 95% probability of detection.

## Discussion

This study provides support for our hypothesis that the behaviour and ecology of the Saharan cheetah is severely constrained by the harsh desert environment, supporting our predictions that Saharan cheetah are: (P1) nocturnal; (P2) potentially wide-ranging; and (P3) occur at very low densities relative to cheetah in savannah environments. The study provides evidence of presence of cheetah in the Ahaggar, with cheetah being detected at 32.5% of camera trap locations across both surveys. It also shows for the first time, to our knowledge, that camera trap methodologies can be used to provide density estimates for such a wide-ranging and low density species.

Nearly all captures of cheetah occurred during darkness. While these data were drawn from a limited number of individuals (five in total across both surveys), and it is possible that activity obtained by camera traps is not representative of the activity cycles of cheetah overall, the lack of captures in daylight hours was very marked, and captures were most numerous in the middle of the night, not during the crepuscular period around dawn and dusk. There was also no evidence that activity was linked to the lunar cycle, suggesting that nocturnal activity was not linked to the availability of moonlight. This provides the first evidence that Saharan cheetah are likely to be mainly nocturnal, as has been previously reported [47,48]. In many other parts of their range cheetah have been reported as being predominantly diurnal as a strategy to avoid interference competition from larger carnivores, particularly lions (*Panthera leo*) and spotted hyaenas (*Crocuta crocuta*) during the night [49,50]. However nocturnal activity has been reported in the Limpopo province, South Africa, possibly to avoid human persecution [51], while in Moremo Game Reserve, Botswana, nocturnal activity has been linked to the lunar cycle, where cheetah are more likely to be active on moonlit nights [52]. In the Ti-n-hağğen region lions and spotted hyaenas are absent and there are reports of occasional persecution of cheetah by people, hence both factors may contribute to a shift to nocturnal behaviour, enabling cheetah to avoid both people and the heat of the day.

Densities of cheetah in the extreme arid environment of Ahaggar were substantially lower than in more productive ecosystems, with densities one order of magnitude lower than those recorded in Namibia [53] and Kruger National Park [54], and up to two orders of magnitude lower than the densities recorded in the highly productive Serengeti ecosystem ([55]; see Table 1). The full MMDM has provided better estimates of true density in studies of other large carnivores where camera-trap density estimates have been independently calibrated. Full MMDM density, averaging 0.23 individuals per 1,000km^2^ over the two surveys, is substantially lower than that recorded for any other African large carnivore, such as lion, leopard and spotted hyaena. There was also evidence that home ranges may be large, with a conservative minimum estimate of 1,583km^2^ which is as large as some of the biggest *annual* home ranges documented elsewhere [56], even though measured only over a 2–3 month period.

Unsurprisingly, considering the very low densities of cheetah in the Saharan environment, abundance estimates have a high coefficient of variation of 27–36%. The survey conducted was close to the limit of what was feasible in the harsh terrain of the Algerian Central Sahara. Access to the area is now strictly limited due to security concerns prevailing in the Sahara-Sahel region, and hence it is unlikely that these estimates will be improved upon for the time being. Though precision of abundance estimates was not high, it is, nevertheless, believed that the low density figures obtained are realistic, given scarce prey availability and the harsh Saharan environment. The consistency of abundance estimates between 2008 and 2010 gives further confidence in their validity.

There was evidence of heterogeneity in capture probabilities from the fit of the mark recapture models to our capture histories of individual cheetah. Cheetah have been previously shown to be highly heterogeneous in detectability elsewhere [46,57]. In our surveys, two solitary males were frequently recaptured during both surveys at sites that stretched across a large proportion of our survey area, and hence their ranges are likely to overlap extensively within our survey area, whereas the other individuals (two in 2008 and one in 2010) were sighted at only a single site and only in a single survey. Other sources of heterogeneity may arise due to differences in status of individuals (e.g. if they were territorial or non-territorial), or in their use of sites where camera traps are placed. In both our surveys a preponderance of males were trapped in our cameras, which suggests that the sites chosen for our cameras, usually at trees, may have been more likely to attract males than females [58]. Sequences of photos from our captures clearly show male behaviours associated with territoriality, including urine marking a tree, clawing and defecating on *Tamarix* branches, suggesting that these behaviours may play a role in the Ahaggar. A preference by males for camera trap sites due to these behaviours could have resulted in underestimation of density in Table 3. However, as sex could not be determined for one animal in each of the 2008 and 2010 surveys, it is possible females were also captured. In the second survey in 2010, temporal variation was also a component of the best fit model to the cheetah capture histories. This could be driven, for example, by temporary vacation of the survey area by cheetah, possibly seeking potential prey or mates.

We used our survey data to develop recommendations for future surveys. Information on density and presence is key for monitoring critically endangered populations, since decision makers need to know both the extent of the distribution of populations and overall abundance, and to be able to reliably estimate trends, in order to best target conservation management interventions [59]. The pattern of accumulation of individuals was very different in both surveys, but was long enough to conclude that the survey effort used was not excessive for estimation of abundance. The logistical constraints of working in an extreme and remote environment, in the face of additional security challenges, make it likely to be impractical to increase survey effort beyond that used here. However, survey effort could be substantially reduced to 868–992 camera trap days if the aim of the survey is solely to detect presence or absence. This could be achieved with 95% probability either by using 40 cameras over 23–24 days, or by using 14–16 cameras over a 2–3 month survey. A similar figure of 1000 camera trap days was predicted as necessary to establish presence or absence of tigers (*Panthera tigris*), where densities could range between 0.4–0.7 tigers/100km^2^ [60] – a density substantially higher than that found for the Saharan cheetah. This study also demonstrated that the probability of detection increases markedly with daily travel distance, and this may be substantially higher for cheetah in desert environments than for tigers in forest. It is also possible that placing cameras close to rare and important resources, such as trees, as in this survey, increases overall likelihood of detection.

The Saharan cheetah persists in the Ahaggar in the face of substantial threats. Two antelope species have already been extirpated from this area, addax (*Addax nasomaculatus*) and dama gazelle (*Nanger dama*); whereas the remaining ungulates, dorcas gazelle and Barbary sheep are hunted at night using spotlights from vehicles ([22]; F.B. pers. obs.). Dorcas gazelle are still relatively widespread in the area, and are likely to be a key prey species for cheetah [61], but it is likely that Addax and dama gazelle may have historically constituted an important food source [62]. Cape hares are also relatively abundant. More surveys are urgently needed across the region to identify other important areas for Saharan cheetah conservation.

The Saharan cheetah has the potential to become a key flagship species for the region. Cheetah have a relatively large body mass and forward facing eyes, characteristics identified as predicting flagship appeal [63]. Such flagship species have been demonstrated to be effective at attracting global attention, and helping to secure resources necessary for their conservation [64]. The presence of charismatic flagship species is also correlated with the sustainability of ecotourism programs, with consequent economic and conservation benefits [65]. Species able to fulfil the role of flagships are generally charismatic and able to mobilise public support and interest [66]. Mountain gorillas (*Gorilla beringei*), golden snub-nosed monkeys (*Rhinopithecus roxellana)* and Komodo monitor lizards (*Varanus komodoensis*) are examples of effective flagships, which have attracted sufficient economic income from tourists to ensure better conservation of these species and their habitats [66–68].

The Saharan cheetah is also one of the widest ranging carnivores, and hence requires large areas for its conservation [69], making it a useful proxy for conservation at the scale of the ecosystem or wider landscape. Asiatic cheetah (*Acinonyx jubatus venaticus*) have successfully been used as flagships in Iran (http://www.wildlife.ir) where, even though they are rarely seen, the presence of this scarce and wide-ranging species is a focus of national pride, and serves as symbolic evidence of wilderness and conservation value, attracting international conservation resources. In Botswana cheetah are also used as a flagship species (http://cheetahbotswana.com) where they attract ecotourism and consequent economic benefits to the government and to local communities. The Saharan cheetah has potential to attract conservation attention, as a critically endangered subspecies, and provide added appeal to visitors to the Saharan region. Until recently, the Ahaggar attracted numerous tourists, lured by the beauty of the desert landscape. Walking, camel and 4×4 vehicle tours have been popular in desert terrain, and the presence of the cheetah can be marketed as providing added value to such destinations. Other large but elusive cats, such as the snow leopard (*Panthera uncia*), have successfully provided a focus for visitors and a market for locally produced handicrafts [70]. Such strategies can be extremely effective at securing economic benefits and political will for conservation, even where there is a low chance of observing flagship species directly.

In conclusion this study 1) demonstrates that the Saharan cheetah is likely to be ecologically and behaviourally constrained in the Sahara; 2) shows that the subspecies occurs at one of the lowest large carnivore densities previously recorded in Africa; 3) demonstrates the applicability of camera traps for surveying wide-ranging low density species in arid environments and provides guidelines for future surveys; and 4) demonstrates the value of the Ahaggar Cultural Park as a key area for the conservation of the critically endangered Saharan cheetah. More broadly, our study demonstrates the critical contribution of desert environments in sustaining unique and important components of biodiversity, and highlights the challenges in monitoring and managing such biodiversity. The vast landscapes of the Sahara, and the low densities of the species it supports, urgently require new approaches to conservation that are able to work across park and national boundaries. Such approaches need to engage local and citizen support for win-win solutions to benefit both people and biodiversity, if they are to be successful. The Saharan cheetah, which is both charismatic and critically endangered, is a potentially important flagship species which could be used to ‘market’ the Saharan landscape, and attract support at a sufficiently large scale to secure benefits for local communities and to safeguard the ecosystems on which it depends.
